# Measuring psychological pain: psychometric analysis of the Orbach and Mikulincer Mental Pain Scale

**DOI:** 10.1186/s42409-021-00025-8

**Published:** 2021-05-17

**Authors:** Madeline P. Casanova, Megan C. Nelson, Michael A. Pickering, Karen M. Appleby, Emma J. Grindley, Lindsay W. Larkins, Russell T. Baker

**Affiliations:** 1 Medical Education, University of Idaho, 875 Perimeter Drive, Moscow, ID 83844 USA; 2 University of Idaho, 875 Perimeter Drive, Moscow, ID 83844 USA; 3 Idaho State University, 921 South 8th Ave, Pocatello, ID 83209 USA

**Keywords:** Psychological pain, Psychometrics, Confirmatory factor analysis, Invariance testing

## Abstract

**Background:**

Suicide is a public health concern, with an estimated 1 million individuals dying each year worldwide. Individual psychological pain is believed to be a contributing motivating factor. Therefore, establishing a psychometrically sound tool to adequately measure psychological pain is important. The Orbach and Mikulincer Mental Pain Scale (OMMP) has been proposed; however, previous psychometric analysis on the OMMP has not yielded a consistent scale structure, and the internal consistency of the subscales has not met recommended values. Therefore, the primary purpose of this study was to assess the psychometric properties of the OMMP in a diverse sample.

**Methods:**

A confirmatory factor analysis (CFA) on the 9-factor, 44-item OMMP was conducted on the full sample (*n* = 1151). Because model fit indices were not met, an exploratory factor analysis (EFA) was conducted on a random subset of the data (*n* = 576) to identify a more parsimonious structure. The EFA structure was then tested in a covariance model in the remaining subset of participants (*n* = 575). Multigroup invariance testing was subsequently performed to examine psychometric properties of the refined scale.

**Results:**

The CFA of the original 9-factor, 44-item OMMP did not meet recommended model fit recommendations. The EFA analysis results revealed a 3-factor, 9-item scale (i.e., OMMP-9). The covariance model of the OMMP-9 indicated further refinement was necessary. Multigroup invariance testing conducted on the final 3-factor, 8-item scale (i.e., OMMP-8) across mental health diagnoses, sex, injury status, age, activity level, and athlete classification met all criteria for invariance.

**Conclusions:**

The 9-factor, 44-item OMMP does not meet recommended measurement criteria and should not be recommended for use in research and clinical practice in its current form. The refined OMMP-8 may be a more viable option to use; however, more research should be completed prior to adoption.

**Supplementary Information:**

The online version contains supplementary material available at 10.1186/s42409-021-00025-8.

## Introduction

Worldwide, an estimated 1 million individuals die by suicide each year (World Health Organization, 2019). In the USA, suicide ranks as the tenth leading cause of death (Heron, 2019). The rankings are more concerning when assessing causes of death by age group (Heron, 2019): suicide is the second, fourth, and eighth leading cause of death for individuals 10–34, 34–44, and 55–64 years of age, respectively. Additionally, rates of suicide have been dramatically increasing in the USA since 1999 (Stone et al., 2018). Therefore, a better understanding of suicide risk and subsequent prevention efforts continue to be critical.

Although many meanings and motivations behind suicide have been documented (e.g., suffering pain from sickness or old age, political or social peril, stressful life events), the theory of personal agony has continued to receive attention from both clinicians and researchers (Conejero, Olié, Calati, Ducasse, & Courtet, 2018; Seidel, 1995; Verrocchio et al., 2016). Leenaars (1996) wrote, “The enemy of life is [psychological] pain… it is the pain of feeling pain… the fear is that the trauma, the crisis is bottomless—an eternal suffering” (p. 224). The eternal suffering described is frequently heard by clinicians and captured in suicide notes with statements like “I can’t stand the pain any longer” (Goldsmith, Pellmar, Kleinman, & Bunney, 2002). Although psychological pain (PsyPn) is extremely important to understand, the complexity and multifactorial nature of PsyPn has resulted in both conceptual and measurement challenges, thus creating significant gaps in the literature (Meerwijk & Shattell, 2012).

Over the last 100 years, several attempts to conceptualize PsyPn have been made. One of the first accounts can be traced back to Freud (1917), who associated PsyPn with an individual’s feelings of mourning or melancholy following loss. Other researchers later described PsyPn as feelings of suffering, emptiness, and a belief that the future was lost and no hope remained (Frankl, 1992). In the 1990s, the term *psychache* was coined to describe a model of intolerable PsyPn (Shneidman, 1998). Shneidman (1998) believed PsyPn was experienced due to frustrated or thwarted essential needs (e.g., to be loved, to protect one’s image, avoid shame). The lack of essential needs caused individuals to experience a number of negative emotions such as guilt, shame, defeat, and hopelessness and eventually led to a generalized experience of unbearable PsyPn. Subsequently, another model of PsyPn described by Bolger (1999), who labeled PsyPn as emotional pain, proposed that a traumatic event shattered an individual’s personal identity and connection with others. The shattering left intense feelings of emotional pain, which was depicted as brokenness, woundedness, loss of self, feelings of disconnection, and the awareness of one’s own negative attributes (Bolger, 1999).

Other terms, in addition to *psychache* and emotional pain that have also been used to describe PsyPn include suffering (Morse, 2011; Rehnsfeldt & Eriksson, 2004), mental pain (Orbach, Mikulincer, Sirota, & Gilboa-Schechtman, 2003), and psychic pain (Yager, 2015). Literature reviews have been conducted on these terms and researchers argued they all refer to the same concept (Conejero et al., 2018; Meerwijk & Weiss, 2011); therefore, there was a call to unify the terms under the umbrella of “psychological pain” (Meerwijk & Weiss, 2011). The recent unification efforts led to the development of an accepted definition after careful examination of various concepts and models of PsyPn: “a lasting, unsustainable and unpleasant feeling resulting from negative appraisal of an inability or deficiency of the self” (Meerwijk & Weiss, 2011).

With a consensus definition established, there was a need to develop a psychometrically sound instrument to adequately measure PsyPn. Several instruments to measure PsyPn have been proposed; however, each one has limitations and relatively few have undergone necessary psychometric analysis. The Psychological Pain Scale (Shneidman, 1999) requires participants to rate their PsyPn, rate perceived PsyPn of five pictures, identify three feelings prominent in their pain, and write an essay describing their PsyPn. Due to the complexity of the scale, a trained individual is needed to administer and interpret the results, and only modest scale reliability has been found (Leenaars & Lester, 2005). The Psychache Scale (Holden, Mehta, Cunningham, & McLeod, 2001) was developed using constructs from the Psychological Pain Scale, but it eliminated the need for a trained individual to administer the scale. The scale, condensed to 13-items, addressed frequency of PsyPn, but did not capture intensity of pain or the unpleasant or negative feelings associated with PsyPn. The Mee-Bunney Psychological Pain Assessment (Mee et al., 2011) was developed as a brief (i.e., 10-item scale) instrument to measure PsyPn, but the questions did not capture the unpleasant or negative feelings associated with PsyPn. Further, descriptions about scale development or testing of the scale structure were not identified in the literature.

The Orbach and Mikulincer Mental Pain Scale (OMMP) may be a more effective option because it was developed using more contemporary approaches (e.g., grounded theory and content analysis, factor analysis) and addressed some of the constraints associated with the other instruments (Orbach et al., 2003). For example, the OMMP does not require a trained administrator and includes questions that assess both the intensity and dimensions of PsyPn (Orbach et al., 2003). The scale also includes more detailed questions regarding various cognitive and affective components of PsyPn (Pompili, Lester, Leenaars, Tatarelli, & Girardi, 2008). To develop questions for the scale, researchers asked a sample of inpatients and normal individuals (age 15–75) to answer several questions about PsyPn and their experiences with PsyPn (Orbach et al., 2003). The responses to these items were analyzed and formatted into a 220-item scale that was then administered in a new sample of individuals (Orbach et al., 2003). Item analysis, reliability, and factor analysis procedures were conducted, resulting in the final 44-item scale. The factor structure and internal consistency of the scale were then assessed and confirmed in a new sample of Israeli Jewish adults (Orbach et al., 2003). The OMMP includes nine factors: experience of irreversibility, loss of control, narcissistic wounds, emotional flooding, freezing, estrangement, confusion, social distancing, and emptiness (Orbach et al., 2003). The OMMP has been administered in clinical populations (Conrad et al., 2009; Guimarães, Fleming, & Cardoso, 2014; Levi et al., 2008; Reisch et al., 2010; Van Heeringen, Van den Abbeele, Vervaet, Soenen, & Audenaert, 2010), college student samples (Heo, 2008; Orbach et al., 2003), and non-clinical community members (Soumani et al., 2011; Tossani et al., 2019). Researchers have primarily used the OMMP to evaluate relationships between PsyPn and depression, suicidal behavior, and anxiety.

Although assessing PsyPn, particularly between groups, is important for clinicians and researchers alike, instruments that have not undergone psychometric evaluation may not provide adequate, accurate, or reliable results. Thus, attempts to draw meaningful conclusions about scores from the instrument may not be recommended. The steps recommended to establish a psychometrically sound instrument include, but are not limited to (1) assessing the proposed items and scale structure using exploratory factor analysis (EFA), (2) verifying the underlying dimensions and scale structure of the instrument using confirmatory factor analysis (CFA), and (3) assessing measurement invariance and population heterogeneity (Boateng, Neilands, Frongillo, Melgar-Quiñonez, & Young, 2018; Brown, 2014; Kline, 2015). An established instrument will be generalizable and allow clinicians and researchers to adequately measure the constructs intended and reliably compare differences between groups and across time (Brown, 2014; Byrne, 2016; Kline, 2015).

A limited number of studies conducted on the OMMP have examined the psychometrics of the scale. A consistent scale structure using either CFA or EFA methods, however, has not been reported (Supplemental Table 1). For example, Guimarães et al. (2014) found a 5-factor, 24-item solution in a drug addicted sample of respondents. In contrast, Tossani et al. (2019) found a 5-factor, 31-item solution in a non-clinical sample (Supplemental Table 1). Heo (2008) investigated the psychometrics in a Korean sample and US student sample; in the Korean sample, a 5-factor, 21-item solution was found, while a 5-factor, 20-item solution was found in the US student sample (Supplemental Table 1). Although a 5-factor solution was consistent across studies, the factors and items included in the final solutions were not identical (Supplemental Table 1). The inconsistency between samples indicates the theoretical framework of the scale is not well-supported (Brown, 2014; Byrne, 2016; Kline, 2015).

Further, the reported internal consistency of the subscales (i.e., experience of irreversibility, loss of control, narcissistic wounds, and emotional flooding) in the initial scale development work (Orbach et al., 2003) exceed the recommended Cronbach’s alpha value ≥ .90 (Leech, Barrett, Morgan, Barrett, & Morgan, 2014; Streiner, 2003; Supplemental Table 2). The high Cronbach’s alpha values for the subscales may indicate multicollinearity, or redundancy among the items used within the subscales (Brown, 2014; Kline, 2015; Leech et al., 2014; McCrae, Kurtz, Yamagata, & Terracciano, 2011; Streiner, 2003). Similarly, the social distancing subscale was initially reported to have a Cronbach’s alpha of .80; however, the items have not consistently met the recommended ≥ .70 level (Leech et al., 2014; Pesudovs, Burr, Harley, & Elliott, 2007) and the items have been removed from the final scale solution in subsequent research (Guimarães et al., 2014; Heo, 2008; Levinger, Somer, & Holden, 2015; Tossani et al., 2019). Researchers who have used the items have reported alphas that range from .34 to .42 (Gvion et al., 2014; Levi et al., 2008; Levi-Belz, Gvion, Grisaru, & Apter, 2017; Soumani et al., 2011). Thus, a reduction of items and/or subscales may be necessary to create a more parsimonious and psychometrically sound scale (Brown, 2014; Kline, 2015).

Despite the use of the OMMP in practice and research, a complete and robust psychometric analysis of the scale has yet to be completed. There is a need to conduct a CFA to test the hypothesized factor structure of the OMMP, ensuring that the items are indirect measures of the hypothesized latent variables (Brown, 2014; Bryant & Yarnold, 1995). Additionally, the inconsistent psychometrics reported for the scale among different populations indicate the need for invariance testing in a diverse sample to ensure the scale is generalizable and unbiased towards different groups. Therefore, the primary purpose of this study was to assess the psychometric properties of the OMMP in a diverse group of individuals using CFA. Because the model fit did not meet recommended levels, an EFA was conducted to establish a more parsimonious scale structure that was then tested in a rigorous covariance model. The secondary purpose was to conduct invariance testing between age groups, sex, activity classification, activity level, and injury status on the parsimonious scale structure identified.

## Methods

The University Institutional Review Board approved the study and participants provided informed consent prior to beginning the survey. Emerging adults and adult participants (Sigelman & Rider, 2017) were recruited using a combination of convenience and snowball sampling methods (Panacek & Thompson, 2007). Members of the research team utilized personal contacts and social media pages to contact and advertise the study to participants. Additionally, participants were recruited using ResearchMatch (Harris et al., 2012), an online volunteer platform designed to match volunteers with researchers. Participants were able to complete an electronic or paper version of the survey. The electronic survey was developed using Qualtrics Survey Software (Qualtrics Inc., Provo, UT), and the identical paper version of the survey was developed using Microsoft Word. Individuals who completed the electronic version were sent a link to the Qualtrics survey; paper copies were printed and distributed to those who opted to complete it by hand. The survey included the OMMP, a pain questionnaire, psychosocial questionnaires, and a participant demographic questionnaire.

### Participants

A total of 1535 individuals completed the survey. Seventy individuals were missing responses to more than 10% of the OMMP items and were removed from the data set. Three individuals were missing less than 10% of the OMMP; therefore, the missing values for those participants were replaced with the rounded mean for each item. A total of 97 individuals reported scores that indicated univariate (z scores ≥ 3.4) outliers, while an additional 217 reported scores that indicated multivariate outliers (Mahalanobis distance ≥ 68.71); these 314 participants were removed from the data set prior to analysis. A total of 1151 participants, ages 18–95 (mean age = 41.01 ± 16.67), were retained for data analysis. Females accounted for 72.4% (*n* = 833) of the sample, while males accounted for 17.9% (*n* = 206). ResearchMatch participants accounted for 41% (*n* = 473) of the sample (*n* = 473), while social media and personal contacts accounted for 59% of the sample (*n* = 678). Participants were grouped by injury classification, mental health diagnosis, education level, activity level, and activity classification (Table 1).

**Table 1:** Demographic data for the OMMP

Characteristics	*N*	%
Sex		
Male	206	17.9
Female	833	72.4
Prefer not to answer	8	0.7
Unknown	104	9.0
Education		
Some high school, no diploma	2	0.2
High school or GED	38	3.3
Some college, no degree	126	10.9
Associate degree	60	5.2
Bachelor’s degree	281	24.4
Master’s degree	385	33.4
Doctoral degree	133	11.6
Other	21	1.8
Unknown	105	9.1
Mental health diagnosis		
Yes	396	34.4
No	633	55.0
Prefer not to answer	18	1.6
Unknown	104	9.0
Ethnicity		
Caucasian	891	77.4
African American	54	4.7
Hispanic	62	5.4
Asian/Pacific Islander	58	5.0
Other	23	2.0
Unknown	63	5.5
Activity level		
Inactive	179	15.6
Low	410	35.6
Medium	336	29.2
High	125	10.9
Unknown	101	8.8
Athletic classification		
Competitive athlete	32	2.8
Recreational athlete	175	15.2
Occupational athlete	128	11.1
Activities of daily living	118	10.3
No athletic participation	595	61.7
Unknown	101	8.8
Injury status		
Healthy	662	57.5
Acute injury	22	1.9
Sub-acute injury	27	2.3
Persistent injury	110	9.6
Chronic injury	229	19.9
Unknown	101	8.8

### Orbach and Mikulincer Mental Pain Scale

The Orbach and Mikulincer Mental Pain Scale (OMMP) consists of 44 items measuring nine unique factors. Factors include experience of irreversibility (nine items; e.g., the pain will never go away), loss of control (ten items; e.g., I have no control over the situation), narcissistic wounds (five items; e.g., I am rejected by everybody), emotional flooding (four items; e.g., There are strong ups and downs in my feelings), freezing (three items; e.g., I feel paralyzed), estrangement (three items; e.g., I am a stranger to myself), confusion (three items; e.g., I have difficulties in thinking), social distancing (four items; e.g., I don’t feel like talking to other people), and emptiness (three items; e.g., I can’t find meaning in my life). Participants rated each statement using a 5-point Likert scale (1 = strongly disagree, 2 = disagree, 3 = agree to some extent, 4 = agree, 5 = strongly agree).

### Pain questionnaire

To assess physical pain severity, individuals completed the Numerical Pain Rating Scale (NPRS; Hartrick, Kovan, & Shapiro, 2003). The NPRS is used to assess patient self-reported pain severity on a 0–10 scale (0 = no pain, 10 = worst pain possible) for three time points during the past 24 h: current pain, best pain (i.e., lowest pain severity in the past 24 h), and worst pain (i.e., highest pain severity in the past 24 h). The pain scores reported for best, current, and worst were averaged to create a score representative of the patient’s level of pain over 24 h. The NPRS has demonstrated good test-retest reliability (intraclass correlation coefficients ranging from .80 to .99), and high correlations were found between the NPRS and two other pain measures (visual analog scale correlations range from .86 to .99; verbal rating scale = .93), indicating good validity (Alghadir, Anwer, Iqbal, & Iqbal, 2018; Bijur, Latimer, & Gallagher, 2003; DeLoach, Higgins, Caplan, & Stiff, 1998; Hawker, Mian, Kendzerska, & French, 2011; Phan et al., 2012; von Baeyer et al., 2009).

### Psychosocial questionnaires

The Patient Health Questionnaire (PHQ-9) was utilized to assess depression (Spitzer, Kroenke, & Williams, 1999). The PHQ-9 includes 10-items, nine of which correspond with the diagnostic criteria for major depressive disorder. Participants rated each question on a 4-point Likert scale (0 = not at all; 3 = nearly every day), indicating how often each statement had bothered them in the past 2 weeks. The PHQ-9 has reported high reliability and validity to measure presence and severity of depression in both clinical and general populations (Kocalevent, Hinz, & Brähler, 2013; Kroenke, Spitzer, & Williams, 2001; Manea, Gilbody, & McMillan, 2012; Martin, Rief, Klaiberg, & Braehler, 2006). Items were then summed to create a composite score. The PHQ-9 has demonstrated good internal reliability with α ranging from .77 to .87 (Kocalevent et al., 2013; Löwe, Kroenke, Herzog, & Gräfe, 2004; Ślusarska et al., 2019; Urtasun et al., 2019; Villarreal-Zegarra, Copez-Lonzoy, Bernabé-Ortiz, Melendez-Torres, & Bazo-Alvarez, 2019). Construct validity has also been demonstrated by comparing the PHQ-9 to scales of quality of life, life satisfaction, emotional well-being, psychological well-being, and mental health (Keum, Miller, & Inkelas, 2018; Kocalevent et al., 2013); convergent validity has been established by comparing the scale to other measures of depression (Löwe et al., 2004; Maroufizadeh, Omani-Samani, Almasi-Hashiani, Amini, & Sepidarkish, 2019). Additionally, responsiveness, (i.e., the validity of the PHQ-9 across time) has also been established (Löwe et al., 2004). Psychometric properties of the scale were assessed using CFA and multi-group invariance (e.g., sex, age, education level, ethnicity socioeconomic status) techniques; researchers found the model met fit indices and passed invariance criteria, allowing for meaningful group comparisons (Galenkamp, Stronks, Snijder, & Derks, 2017; Keum et al., 2018; Villarreal-Zegarra et al., 2019).

The Self-Compassion Scale (SCS) was utilized to assess self-compassion (Neff, 2003). The SCS includes 26 items to measure six factors: self-kindness (e.g., I’m kind to myself when I’m experiencing suffering), self-judgment (e.g., When times are really difficult, I tend to be tough on myself), common humanity (e.g., I try to see my failings as part of the human condition), isolation (e.g., When I fail at something that’s important to me I tend to feel alone in my failure), mindfulness (e.g., When something upsets me I try to keep my emotions in balance), and over-identification (e.g., When something upsets me I get carried away with my feelings). Participants indicated how often they acted in the manner stated in each of the items using a 5-point Likert scale (1 = almost never; 5 = almost always). Items in each factor were summed to create six subscale scores; items were also summed to create a total score (Neff et al., 2019). The SCS has demonstrated good internal reliability with α ranging from .75 to .81 and test-retest reliability with α ranging from .80 to .88 (Neff, 2003). Psychometric properties of the scale were assessed using CFA and ESEM techniques across 20 samples; excellent fit was found for the six-factor solution (Neff et al., 2019). Additionally, predictive validity has also been demonstrated by comparing the SCS to scales of neuroticism, happiness, optimism, depression, stress, anxiety, and healthier physiological responses to stress (Breines et al., 2014; Finlay-Jones, Rees, & Kane, 2015; Friis, Johnson, Cutfield, & Consedine, 2016; Neff, 2003; Neff, Rude, & Kirkpatrick, 2007).

The Depression Anxiety Stress Scales-21 (DASS-21) was used to assess perceived psychological distress (Lovibond & Lovibond, 1995). The DASS-21 includes 21 items assessing depression (e.g., I couldn’t seem to experience any positive feeling at all), anxiety (e.g., I experienced breathing difficulty), and stress (e.g., I found it hard to wind down). Participants were asked to rate each statement, indicating how much the statement applied to them over the past week using a 4-point Likert scale (0 = did not apply to me at all; 1 = applied to me to some degree, or some of the time; 2 = applied to me a considerable degree, or a good part of the time; 3 = applied to me very much, or most of the time). Items from each subscale were summed to create composite scores, with the cumulative score representing psychological distress. The DASS-21 has demonstrated good internal reliability with α ranging from .73 to .87 (Lovibond & Lovibond, 1995; Osman et al., 2012) and good test re-test reliability with α ranging from .77 to .89 (Asghari, Saed, & Dibajnia, 2008). Convergent validity has been established by comparing the scale to anxiety, depression, and stress scales (Asghari et al., 2008; Bottesi et al., 2015; Lovibond & Lovibond, 1995;Osman et al., 2012 ; Tonsing, 2014), and construct validity of a 3-factor model using EFA and CFA techniques has also been established (Osman et al., 2012; Tonsing, 2014).

### Participant questionnaire

A participant questionnaire was created to collect demographic data including sex, ethnicity, age, highest level of education, physical activity level, diagnosis of a mental illness, and injury status.

### Data analysis

A member of the research team input paper survey responses into Qualtrics. Data was then exported from Qualtrics for analysis into the Statistical Package for Social Sciences Version 26 (SPSS, Inc., Chicago, IL). Missing responses were calculated for the OMMP and individuals missing 4 or more items (i.e., 10%) were removed from the dataset (Kline, 2015). Individuals missing less than 10% of the items (i.e., 3 items or less) were retained, and missing data were replaced with the rounded mean score of the respective item (Kline, 2015). Because the primary purpose was to assess the OMMP, individuals were not excluded if they were missing demographic information or responses to other instruments included in the survey packet. Continuous variables were reported as mean ± SD, and categorical variables were reported as *n*, percentage.

Histograms and skewness and kurtosis values were used to assess for normality of the data. Univariate outliers were removed when the *z*-scores exceeded the cutoff value of |3.3|. Multivariate outliers were removed when Malahanobis distance, identified using a chi-square table with degrees of freedom and *p* value of .01 (Kline, 2015), was exceeded. After assessment of normality and outliers, the full sample was used to conduct a CFA using maximum likelihood estimation. Because model fit did not meet recommended guidelines (Bryant & Yarnold, 1995; Kline, 2015), the full sample was randomly split into two datasets (n1, n2). To identify a more parsimonious solution, an EFA was conducted on sample n1. The solution found during the EFA process was then tested in a more rigorous covariance model approach (Kline, 2015) using sample n2 and further refinement led to the creation of a refined model. A latent variable model was then assessed between the refined OMMP and the original OMMP, to assess the amount of variance accounted for in the new solution. The refined OMMP then underwent multigroup invariance testing. Invariance testing was conducted across sex, age groups, activity classification, activity level, and injury status. Finally, latent variable correlations were performed to assess the relationships between the refined OMMP, the pain questionnaire, and the psychosocial measures.

#### Confirmatory factor analysis

To test the factorial validity of the original 9-factor, 44-item scale, a CFA using maximum likelihood estimation was conducted on the full sample using the Analysis of Moment Structures (AMOS) Version 26 software (IBM Corp., Armonk, NY). In addition to the originally proposed CFA model, two bi-factor CFA models (i.e., SDR, Valence) were fitted to assess for potential method effects of the scale. The SDR (i.e., socially desirable responding) model was a single-bifactor model with a general factor representing SDR and nine group factors (e.g., freezing, emotional flooding). The valence model was a two-bifactor model that included two correlated general factors representing items with a negative valence (42 items) and items with a positive valence (2 items) and nine group factors. The general factors (i.e., SDR, negative valence, positive valence) and all domain factors were uncorrelated, scaled by setting variance to 1.0, and fitted using maximum likelihood estimation. Bi-factor models were assessed to determine if these models provided an improved representation of the data by assessing overall goodness of fit and parameter estimates.

Overall goodness of fit was evaluated by assessing the likelihood ratio statistic (Chi-square or CMIN), Comparative Fit Index (CFI), Tucker-Lewis Index (TLI), Root Mean Square Error of Approximation (RMSEA), and Bollen’s Incremental Fit Index (IFI; Bryant & Yarnold, 1995; Hu & Bentler, 1999; Kline, 2015). Because the chi-square statistic is heavily influenced by sample size, it was not used as a primary assessment of model fit; model fit was deemed acceptable if contemporary criteria were met CFI ≥ .95, TLI ≥ .95, RMSEA ≤ .06, and IFI ≥ .95. Localized areas of strain in the solution were assessed, and the interpretability, size, and statistical significance of the model’s parameter estimates (i.e., factor variances, covariances, and indicator errors) were also reviewed (Brown, 2014).

#### Exploratory factor analysis

EFA using maximum likelihood extraction with direct oblimin rotation was conducted on sample n1. Three criteria were utilized to determine the number of factors retained: (1) factors with an eigenvalue > 1.0, (2) scree plot inflexion point examination, and (3) factors that accounted for more than 5% of the variance (Brown, 2014; Hayton, Allen, & Scarpello, 2004; Leech et al., 2014; Schönrock-Adema, Heijne-Penninga, Van Hell, & Cohen-Schotanus, 2009). Parallel analysis was employed as an additional method to determine the number of factors to retain (O’Connor, 2000). The eigenvalues in the original data set were compared to the randomly ordered data set to guide factor retention.

Assessment of Bartlett’s test for sphericity (< .001) and Kaiser-Meyer Olkin Measure of Sampling Adequacy (≥ .70) were checked for violations (Leech et al., 2014). Following extraction, items were assessed individually and removed one at a time until a parsimonious solution was found. Items were assessed for content and design and removal was guided by commonly accepted recommendations: loading < .40, cross-loading ≥ .30, high bivariate correlations with another item in the scale, poor theoretical or conceptual fit of an item, and/or the item contributed to low internal consistency (Brown, 2014; Leech et al., 2014; Pesudovs et al., 2007; Streiner, 2003). Lastly, Cronbach’s alpha was assessed on each factor and set a priori at ≥ .70 and ≤ .89 (Leech et al., 2014; Morgado, Meireles, Neves, Amaral, & Ferreira, 2018; Pesudovs et al., 2007).

#### Covariance model

The parsimonious solution identified during EFA was then tested using covariance modeling in sample n2. The same goodness-of-fit criteria that were utilized for the initial CFA were also used to assess acceptability of model fit for the covariance model (Brown, 2014; Kline, 2015). In addition, modification indices, factor loadings, and correlations between variables were observed. To determine if the refined version of the scale explained an acceptable amount of variance (*r* ≥ 0.90; *R*^2^ = 0.81; Raes, Pommier, Neff, & Van Gucht, 2011) a correlational analysis was conducted on the scores of the OMMP and the refined OMMP.

#### Invariance testing

Using the full sample, the refined model was then subjected to multigroup invariance testing. AMOS (IBM Corp., Armonk, NY) software was utilized to perform the analysis across sex (i.e., male, female), age (i.e., emerging adults, adults), injury status (i.e., injured, healthy), activity level (i.e., inactive/low, moderate/high), and activity classification (i.e., individuals who participated in athletic activity, individuals who did not participate in athletic activity). Invariance testing is necessary to determine if the association between the underlying latent constructs (e.g., PsyPn, confusion, loss of control, narcissistic wounds) and their respective items are stable and approximately equal across groups (Brown, 2014; Byrne, 2016; Kline, 2015; Van De Schoot, Schmidt, De Beuckelaer, Lek, & Zondervan-Zwijnenburg, 2015). An invariant model ensures individuals of different groups are interpreting the survey items and meanings of the items similarly, regardless of group membership (e.g., male or female), which confirms scores from the instrument truly correspond with the underlying constructs and are not due to group-specific attributions. Instrument invariance is necessary to ensure the scale can be used to compare hypothesized group differences (e.g., do females report higher mean scores on PsyPn than males).

Invariance testing involves a set of hierarchical steps with increasing levels of constraint (Brown, 2014; Byrne, 2016; Gregorich, 2006; Kline, 2015). First, individual CFAs by subgroup category (e.g., male and female, injured and healthy) were conducted, ensuring the operationalization of the construct and factors (e.g., confusion, irreversibility, social distancing) were present. Following individual CFAs, the model then underwent configural, metric, and scalar invariance. Configural invariance places both groups in the same model and ensures the same factors have identical items across groups (e.g., emptiness has three items with substantial loadings in both males and females). The configural model serves as the baseline to which all subsequent models are then tested against (i.e., CFI_diff_ and X2_diff_ are calculated by determining the difference between the configural model values and the model being tested). Metric invariance tests if the factor loadings are equal across groups; thus, invariance at this step would ensure the meanings of the common factors are similar across groups. Finally, scalar invariance ensures that item intercepts are equal across groups, implying the means are not driven or contaminated by outside factors (e.g., cultural norms, group specific attributes). Therefore, scalar invariance allows for means of the latent variables to be meaningfully compared across groups. If the model met metric invariance requirements, equal variances were assessed; if the model met scalar invariance requirements, equal mean models were assessed. Model fit was compared using the CFI difference test (CFI_DIFF_) and the chi-square difference test (*χ*^2^_DIFF_), with a *p* value cutoff of 0.01 (Brown, 2014; Byrne, 2016). The CFI_DIFF_ test held greater weight in decisions regarding model fit because the *χ*^2^_DIFF_ test is sensitive to sample size (Brown, 2014; Kline, 2015). Therefore, if a model exceeded the *χ*^2^_DIFF_ test but met the CFI_DIFF_ test, invariance testing proceeded.

#### Correlation models

AMOS (IBM Corp., Armonk, NY) Version 26 was used to assess latent variable correlations between the second order refined OMMP and psychosocial questionnaires (i.e., PHQ-9, SCS, DASS-21). Additionally, correlations were assessed between the refined OMMP and subscales of the DASS-21 and the average NPRS pain score.

## Results

### Confirmatory factor analysis Orbach and Mikulincer Mental Pain Scale

**Fig. 1:**
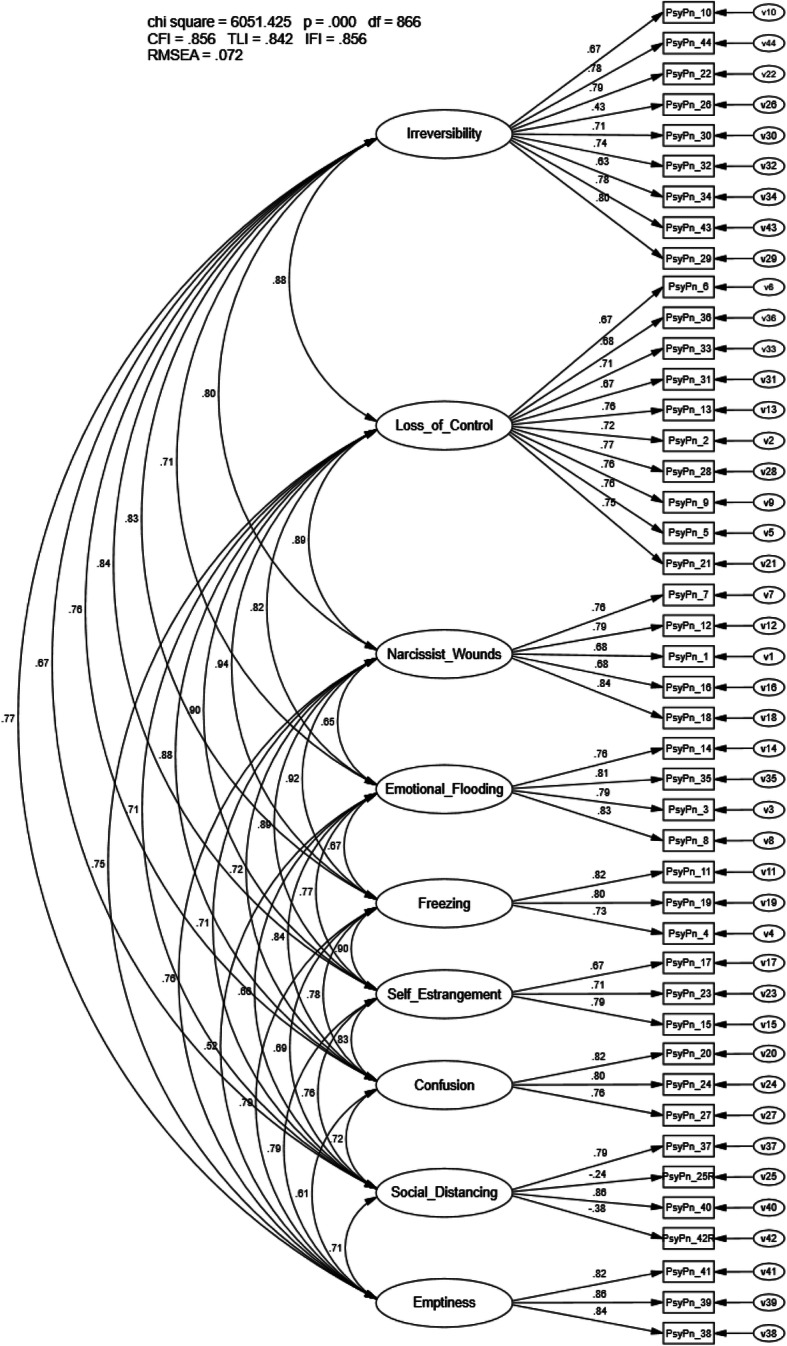
Confirmatory factor analysis Orbach and Mikulincer Mental Pain Scale

The CFA of the 9-factor, 44-item OMMP goodness-of-fit indices did not meet recommended values (CFI = .856, TLI = .842, RMSEA = .072, IFI = .856, *p* < .001; Fig. 1). Factor loadings were significant and ranged from − .24 to .86; however, correlations between first-order latent variables (e.g., irreversibility, emptiness) were high, ranging from *r* = .52 to *r* = .94 (Supplemental Table 3) and modification indices suggested several meaningful cross-loadings were present. Neither of the bi-factor CFA models (i.e., SDR, Valence) provided a substantial improvement for representation of the data and fit indices did not meet recommended values (SDR bi-factor CFI = .856, TLI = .856, RMSEA = .069, IFI = .868, *p* < .001; valence bi-factor CFI = .870, TLI = .857, RMSEA = .069, IFI = .870, *p* < .001). Of note, a general factor may be present; however, a pattern to indicate it is related to SDR was not present. Overall, both bi-factor models had inadequate fit, suggesting the data did not support the models.

Therefore, the dataset was randomly split into two equal samples (n1 = 576, n2 = 575) for further analysis because of possible multicollinearity between first-order latent variables and overall model fit failing to meet recommended values. Sample n1 was used for EFA procedures, while sample n2 was used to assess fit of the refined solution in a covariance model.

### Exploratory factor analysis

Initial EFA of the OMMP in sample n1 extracted four factors with eigenvalues over 1 that accounted for 60.35% of the variance (Supplemental Table 4). Parallel analysis also indicated that four factors should be retained; however, the eigenvalue for the fourth factor narrowly surpassed the random data eigenvalue (Supplemental Table 5). Following extraction, item loadings, cross-loadings, and analysis of item content were assessed; 14 items that had low loadings, substantial cross-loadings, or poor conceptual fit were eliminated. As the process continued, an additional 21 items (35 items in total) were removed due to low loadings, high cross-loadings, inflated Cronbach’s alpha levels, high inter-item correlation values, or lack of conceptual fit. Item removal resulted in a 3-factor, 9-item refined OMMP (i.e., OMMP-9) that accounted for 75.38% of the variance, contained items with loadings ≥ .43, and had Cronbach’s alphas ranging from .767 to .856 (Supplemental Table 6).

Factor 1 contained items 44, 29, and 32 that tapped into the belief that the experience is perpetual and retained the original label “Experience of Irreversibility.” Factor 2 contained items 8, 35, and 14 and tapped into experiencing extreme emotions and feelings; it retained the original label “Emotional Flooding.” Factor 3 contained items 7, 1, and 16 and tapped into an individual’s negative self-belief regarding social relationships and retained the original label “Narcissistic Wounds.”

### Covariance model refined OMMP-9

**Fig. 2:**
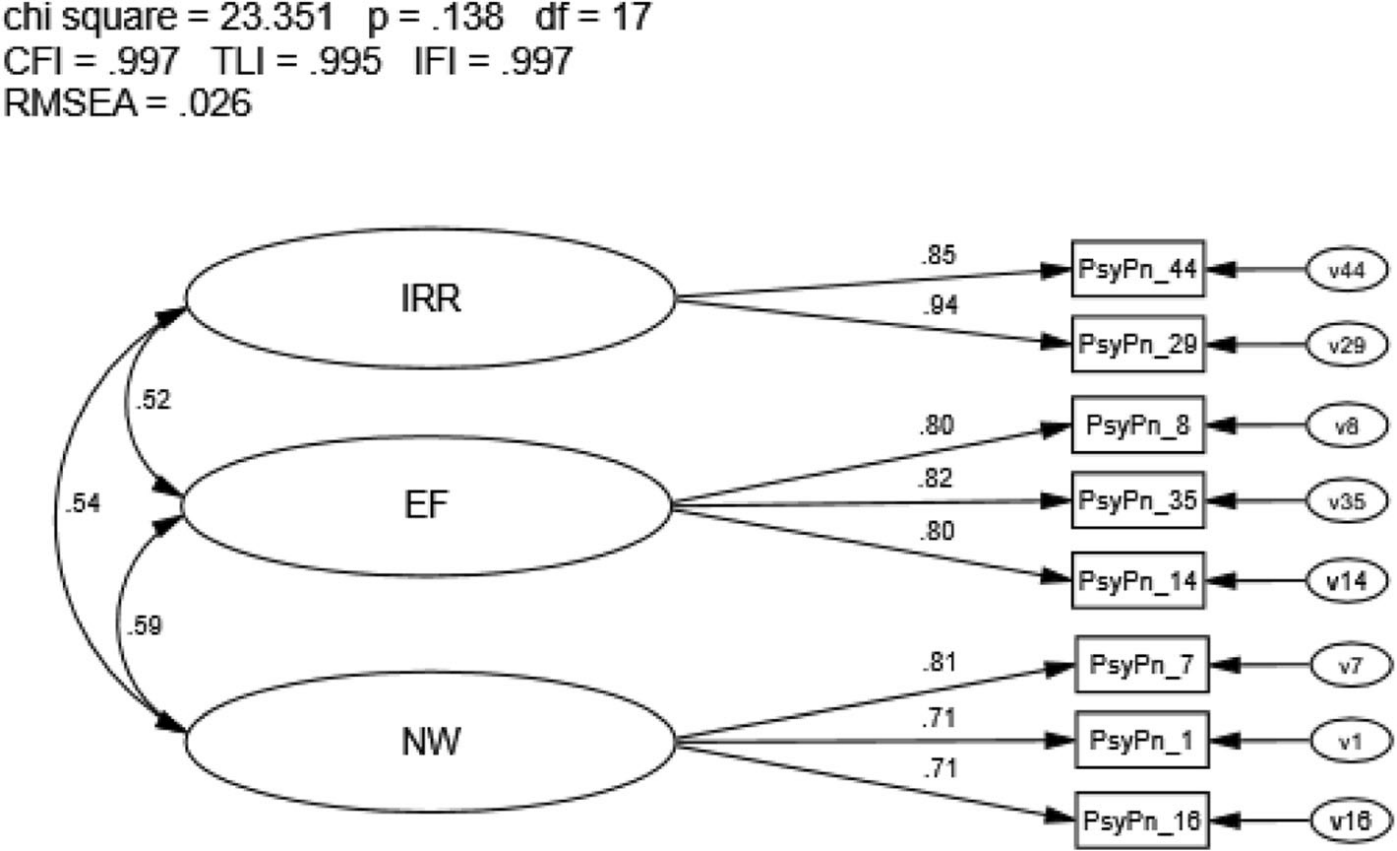
Covariance model OMMP-8

The covariance model of the OMMP-9 in sample n2 had improved model fit (Supplemental Figure 1) with almost all goodness of-fit indices meeting recommended values (CFI = .968, TLI = .952, RMSEA = .076, IFI = .968, *p* < .001; Hu & Bentler, 1999; Kline, 2015). Factor loadings were significant and ranged from .68 to .89, while the correlations between first-order latent variables (e.g., irreversibility, emptiness) were improved, ranging from *r* = .55 to .59. Modification indices indicated there was one item with meaningful cross-loadings; therefore, further refinement of the model was performed. Item 32 was removed, which resulted in a 3-factor, 8-item scale (i.e., OMMP-8) with all model fit indices exceeding recommended values (CFI = .997, TLI = .995, RMSEA = .026, IFI = .997, *p* = .138; Fig. 2). Factor loadings were significant, ranging from .71 to .94, and moderate correlations between first-order latent variables (range = .52 to .59) were present.

Participant scores for the original 44-item OMMP were highly correlated (*r* = .925, *R*^2^ = .856) with participant scores from the OMMP-8. The high correlation value indicated participant responses on the OMMP-8 explained an acceptable amount of variance in responses on the original OMMP.

### Invariance testing of refined OMMP-8

#### Invariance analysis for mental health diagnosis

Of the 1,151 individuals in the full sample, 1029 (89.4%) reported history of mental health diagnosis (yes = 396, no = 633) and were used for analysis. The initial model (i.e., equal form) met all model fit indices (CFI = .988; *χ*^2^ = 78.56; RMSEA = .036; Supplemental Table 7). The metric model (i.e., equal loadings) passed both the CFI_DIFF_ test (CFI = .988) and the *χ*^2^_DIFF_ test (*χ*^2^ = 83.30). Because the metric model was invariant between groups, examination of the equal latent variable factors was warranted. The equal factor variance model passed the CFI_DIFF_ test (CFI = .978) and only slightly exceeded the *χ*^2^_DIFF_ test (*χ*^2^ = 122.23), indicating variances of the latent variables were equal between groups. The scalar model (i.e., equal intercepts) passed both the CFI_DIFF_ test (CFI = .985) and the *χ*^2^_DIFF_ test (*χ*^2^ = 100.20). Because the scalar model was invariant between groups, examination of the latent mean model was warranted. The equal latent means model did not pass the CFI_DIFF_ test (CFI = .956) or the *χ*^2^_DIFF_ test (*χ*^2^ = 129.60), indicating there were differences in means between groups. When means were not constrained to be equal, the group that reported a current or past mental health diagnosis exhibited substantially higher levels of PsyPn across all three constructs (i.e., experience of irreversibility, emotional flooding, and narcissistic wounds) than the group who reported no mental health diagnosis.

#### Invariance analysis for sex

Of the 1151 individuals in the sample, 1,039 (90.3%) reported sex (male = 206, female = 833) and were used for analysis. The initial model (i.e., equal form) met all model fit indices (CFI = .987; *χ*^2^ = 84.15; RMSEA = .038; Supplemental Table 8). The metric model (i.e., equal loadings) passed both the CFI_DIFF_ test (CFI = .988) and the *χ*^2^_DIFF_ test (*χ*^2^ = 86.61). Because the metric model was invariant between groups, examination of the equal latent variable factors was warranted. The equal factor variance model passed both the CFI_DIFF_ test (CFI = .988) and the *χ*^2^_DIFF_ test (*χ*^2^ = 89.75), indicating variances were equal between groups. The scalar model (i.e., equal intercepts) passed both CFI_DIFF_ test (CFI = .985) and the *χ*^2^_DIFF_ test (*χ*^2^ = 101.13). Because the scalar model was invariant between groups, examination of the latent mean model was warranted. The equal latent means model passed the CFI_DIFF_ test (CFI = .978) and slightly exceeded the *χ*^2^_DIFF_ test (*χ*^2^ = 48.53), indicating there were no differences in means between groups.

#### Invariance analysis for injury status

Of the 1,151 individuals in the sample, 1050 (91.2%) reported injury status (healthy = 662, injured = 388) and were used for analysis. The initial model (i.e., equal form) met all model fit indices (CFI = .993; *χ*^2^ = 59.49; RMSEA = .027; Supplemental Table 9). The metric model (i.e., equal loadings) passed both the CFI_DIFF_ test (CFI = .994) and the *χ*^2^_DIFF_ test (*χ*^2^ = 63.28). Because the metric model was invariant between groups, examination of the equal factor variance model was warranted. The equal factor variance model did not pass the CFI_DIFF_ test (CFI = .961) or the *χ*^2^_DIFF_ test (*χ*^2^ = 190.45), indicating variances were not equal between groups. Examination of the variances when not constrained to be equal indicated that the injured group exhibited substantially more variance on the latent variable “Experience of Irreversibility” than the healthy group.

The scalar model (i.e., equal intercepts) passed both the CFI_DIFF_ test (CFI = .993) and the *χ*^2^_DIFF_ test (*χ*^2^ = 72.40). Because the scalar model was invariant between groups, examination of the latent mean model was warranted. The equal latent means model did not pass the CFI_DIFF_ test (CFI = .954) or the *χ*^2^_DIFF_ test (*χ*^2^ = 222.23), indicating there were differences in means between groups. When means were not constrained to be equal, the injured group reported higher levels of PsyPn in all three constructs (i.e., experience of irreversibility, emotional flooding, and narcissistic wounds) than the healthy group.

#### Invariance analysis for age

Of the 1151 individuals in the sample, 1047 (91.0%) reported age and were used for analysis. Individuals were grouped according to human developmental literature (Sigelman & Rider, 2017): emerging adulthood (ages 18–25; *n* = 211), early adulthood (ages 26–40; *n* = 388), middle adulthood (ages 41–65; *n* = 334), late adulthood (ages 66+; *n* = 114). The configural model (i.e., equal form) met all model fit indices (CFI = .993; *χ*^2^ = 96.16; RMSEA = .020; Supplemental Table 10). The metric model (i.e., equal loadings) passed both the CFI_DIFF_ test (CFI = .993) and the *χ*^2^_DIFF_ test (*χ*^2^ = 244.59). Because the metric model was invariant between groups, examination of equal factor variance model was warranted. The equal factor variance model did not pass the CFI_DIFF_ test (CFI = .964) or the *χ*^2^_DIFF_ test (*χ*^2^ = 134.47), indicating variances were not equal between groups. Examination of the variances when not constrained to be equal indicated that the group variances differed across the three latent variables. The middle adulthood group exhibited substantially more variance on the latent variable “Experience of Irreversibility,” and the late adulthood group exhibited substantially less variance on the latent variables “Emotional Flooding” and “Narcissistic Wounds.”

The scalar model (i.e., equal intercepts) slightly exceeded the CFI_DIFF_ test (CFI = .982); however, it passed the *χ*^2^_DIFF_ test (*χ*^2^ = 72.40) and met an additional recommendation of RMSEA_DIFF_ test < .015 (RMSEA = .026; Chen, 2007), indicating the model was invariant between groups. Therefore, examination of the latent mean model was warranted. The equal latent means model did not pass the CFI_DIFF_ test (CFI = .940) or the *χ*^2^_DIFF_ test (*χ*^2^ = 341.65), indicating there were differences in means between age groups. When means were not constrained to be equal, the late adulthood group reported lower levels of PsyPn in latent constructs “Emotional Flooding” and “Narcissistic Wounds”, while the middle adulthood group exhibited higher levels of PsyPn in latent construct “Experience of Irreversibility” than the emerging and early adulthood groups.

#### Invariance analysis for activity level

A total of 1,050 (91.2%) individuals in the sample reported activity level (inactive/low = 589, moderate/high = 461) and were used for analysis. The initial model (i.e., equal form) met all model fit indices (CFI = .995; *χ*^2^ = 50.94; RMSEA = .022; Supplemental Table 11). The metric model (i.e., equal loadings) passed both the CFI_DIFF_ test (CFI = .996) and the *χ*^2^_DIFF_ test (*χ*^2^ = 55.33). Because the metric model was invariant between groups, examination of the equal factor variance model was warranted. The equal factor variance model did not pass the CFI_DIFF_ test (CFI = .980) or the *χ*^2^_DIFF_ test (*χ*^2^ = 117.11), indicating variances were not equal between groups. Examination of the variances when not constrained to be equal indicated the inactive/low group exhibited substantially more variance on the latent variable “Experience of Irreversibility” than the healthy group.

The scalar model (i.e., equal intercepts) passed both the CFI_DIFF_ test (CFI = .995) and the *χ*^2^_DIFF_ test (*χ*^2^ = 62.75). Because the scalar model was invariant between groups, examination of the latent mean model was warranted. The equal latent means model did not pass the CFI_DIFF_ test (CFI = .974) or the *χ*^2^_DIFF_ test (*χ*^2^ = 145.27), indicating there were differences in means between groups. When means were not constrained to be equal, the inactive/low group reported higher levels of PsyPn in all three constructs (i.e., experience of irreversibility, emotional flooding, and narcissistic wounds) than the moderate/high activity group.

#### Invariance analysis for activity classification

A total of 1050 (91.2%) individuals in the sample reported activity classification (i.e., if they engaged in athletic, recreational, or occupational activities that require physical skills and use strength, power, endurance, speed, flexibility, range of motion, or agility at least 3 days per week) and were used for analysis (athletic activity = 455, no athletic activity = 595). The initial model (i.e., equal form) met all model fit indices (CFI = .991; *χ*^2^ = 68.13; RMSEA = .031; Supplemental Table 12). The metric model (i.e., equal loadings) passed both the CFI_DIFF_ test (CFI = .991) and the *χ*^2^_DIFF_ test (*χ*^2^ = 72.16). Because the metric model was invariant between groups, examination of the equal factor variance model was warranted. The equal factor variance model slightly exceeded the CFI_DIFF_ test (CFI = .980) and the *χ*^2^_DIFF_ test (*χ*^2^ = 116.38) however passed the RMSEA_DIFF_ < .015, indicating variances were equal between groups.

The scalar model (i.e., equal intercepts) passed both the CFI_DIFF_ test (CFI = .990) and the *χ*^2^_DIFF_ test (*χ*^2^ = 82.58). Because the scalar model was invariant between groups, examination of the latent mean model was warranted. The equal latent means model did not pass the CFI_DIFF_ test (CFI = .972) or the *χ*^2^_DIFF_ test (*χ*^2^ = 154.13), indicating there were differences in means between groups. When means were not constrained to be equal, the group who did not participate in athletic activity reported higher levels of PsyPn in all three constructs (i.e., experience of irreversibility, emotional flooding, and narcissistic wounds) than the group who did participate in athletic activity.

### Correlational analysis

There were significant correlations between the OMMP-8 and the latent variable models of the PHQ-9 (*r* = .90, *R*^2^ = .81, *p* < .001), SCS (*r* = -.85, *R*^2^ = .72, *p* < .001), and DASS-21 (*r* = .86, *R*^2^ = .74, *p* < .001). Correlations were also significant between the OMMP-8 higher-order model and the subscales of the DASS-21 (depression *r* = .84, *R*^2^ = .71, *p* < .001; stress *r* = .74, *R*^2^ = .54, *p* < .001; anxiety *r* = .67, *R*^2^ = .45, *p* < .001) and the average NPRS pain score (*r* = .56, *R*^2^ = .32, *p* < .001).

## Discussion

Suicide is a public health concern, with an estimated one million individuals dying by suicide each year worldwide (World Health Organization, 2019). Several theories behind the meaning and motivation of suicide have been proposed; however, individual PsyPn is believed to be a contributing factor and has continued to be assessed (Conejero et al., 2018; Seidel, 1995; Verrocchio et al., 2016). Therefore, establishing a psychometrically sound tool to adequately measure PsyPn may be valuable. Previous psychometric analysis on the OMMP has not yielded a consistent scale structure (Guimarães et al., 2014; Heo, 2008; Tossani et al., 2019), and the internal consistency of the subscales has not met recommended values (Guimarães et al., 2014; Gvion et al., 2014; Heo, 2008; Levi et al., 2008; Levi-Belz et al., 2017; Soumani et al., 2011). Therefore, the primary purpose of this study was to assess the psychometric properties of the OMMP in a diverse sample.

The CFA of the original 9-factor, 44-item OMMP did not meet recommended model fit indices. Therefore, an EFA was conducted to establish a more parsimonious scale (i.e., OMMP-9) structure. The OMMP-9 was then tested in a covariance model and refined further to create the OMMP-8. The OMMP-8 was then subjected to invariance testing between age groups, sex, activity classification, activity level, and injury status. The findings of our study suggest that the 9-factor, 44-item OMMP does not meet recommended measurement criteria and should not be recommended for use in research and clinical practice. The refined OMMP-8 may be a more viable option to use; however, more research should be completed prior to adoption.

### Confirmatory factor analysis

The original 9-factor scale structure was not supported in our study due to poor model fit indices and high latent variable correlations indicating many sub-dimensions were not measuring unique constructs. Our findings are consistent with previous research which has failed to identify a consistent scale structure (Guimarães et al., 2014; Heo, 2008; Tossani et al., 2019). Correlations between first-order latent variables were moderate to very high (ranged from .52 to .94), indicating multicollinearity between factors and poor discriminant validity. Modification indices also suggested there were items with meaningful cross-loadings (i.e., items measured several factors), further suggesting multicollinearity and a lack of distinction between factors. The inconsistent factor structure, poor model fit, validity concerns (i.e., factorial and discriminant), and possible multicollinearity provide evidence that the scale should not be used in its current format. Thus, scale refinement using alternate model generation was warranted to determine whether a psychometrically sound version could be identified using the current items.

### Refined OMMP psychometric analysis

An EFA was conducted in a calibration sample (i.e., n1) and a 9-item, 3-factor solution (i.e., OMMP-9) emerged. The nine items represented three of the original nine factors: three items from “Experience of Irreversibility,” three items from “Emotional Flooding,” and three items from “Narcissistic Wounds.” The OMMP-9 was then subjected to covariance modeling procedures using the validation sample (i.e., n2). Although the model had improved fit, modification indices suggested further refinement could improve model fit: item 32 (i.e., something in my life was damaged forever) was therefore removed from the model due to meaningful cross-loadings. The final model (i.e., OMMP-8) retained eight of the original items and represented three distinct factors (i.e., Experience of Irreversibility, Emotional Flooding, and Narcissistic Wounds). The retained factors capture the essence of the definition (i.e., “a lasting, unsustainable and unpleasant feeling resulting from negative appraisal of an inability or deficiency of the self”; Meerwijk & Weiss, 2011).

Although the OMMP-8 only retained 18% of the questions from the original scale, participant responses were highly correlated (*r* = .925) with the original OMMP. Participant scores on the OMMP-8 accounted for a substantial amount of the variance (*r*^2^ = .856) in the responses to the original 44-item OMMP (Raes et al., 2011). On average, participant scores for the OMMP-8 (mean = 1.99) were similar to those found in previous non-clinical samples (Gvion et al., 2014; Nahaliel et al., 2014; Tossani et al., 2019) and were lower than those found in clinical populations (Guimarães et al., 2014;Gvion et al., 2014 ; Levi et al., 2008 ; Nahaliel et al., 2014).

The 3-factor structure identified in our sample was not consistent with previous research that identified 5-factor structures in their samples (Guimarães et al., 2014; Heo, 2008; Orbach et al., 2003). The items included in the scale were also not consistent except for items 7, 14, 35, and 8 (Guimarães et al., 2014; Heo, 2008; Orbach et al., 2003; Tossani et al., 2019). Additionally, the only factor that has emerged across the five studies was “Emotional Flooding” (Guimarães et al., 2014; Heo, 2008; Orbach et al., 2003; Tossani et al., 2019). Although our study found a parsimonious model, more research should be done to ensure the scale structure identified is replicated in subsequent samples.

### Refined OMMP-8 invariance testing

The OMMP-8 passed multigroup measurement invariance criteria for all group classifications: sex, injury status, activity level, mental health diagnosis, age, and activity classification. Thus, researchers can use the OMMP-8 to examine differences in PsyPn among these groups through a comparison of group mean scores. We did not identify group mean differences in PsyPn between males and females or between individuals who were healthy and injured on the OMMP-8. Our results are similar to previous research that did not identify differences between males and females in the subscales of “Irreversibility” and “Narcissistic Wounds” (Tossani et al., 2019); however, our results also differ with previous research that identified group mean differences in “Emotional Flooding” between males and females (Tossani et al., 2019). Although no differences were identified in our sample, subsequent research should continue to assess for differences as previous literature has indicated females exhibit higher levels of rumination which contribute to higher rates of depression (Broderick & Korteland, 2002; Johnson & Whisman, 2013).

Group mean differences in PsyPn were identified between individuals with and without a current or past mental health diagnosis. Our results indicate individuals with a past or current mental health diagnosis exhibited substantially more PsyPn than those who did not have a past or current mental health diagnosis. This finding is consistent with previous research (Gvion et al., 2014; Levi et al., 2008; Nahaliel et al., 2014) and provides further evidence of content validity for the OMMP-8 (Kline, 2015). Clinical populations have reported higher levels of PsyPn and previous researchers have found that scores on subscales of the OMMP can distinguish individuals based on the likelihood they will engage in a high-risk suicide attempt (Levi-Belz et al., 2017) or if they have suicidal tendencies (Nahaliel et al., 2014). Although these measures were not assessed in the present study, future research should assess the ability of the OMMP-8 to distinguish individuals with and without high suicide risk.

Group differences in variances and means for PsyPn were also found between activity levels. Individuals who were classified as being inactive or engaging in low physical activity had substantially more variance (i.e., dispersion) in their responses and exhibited substantially more PsyPn than those who were active. Similarly, those who did not engage in athletic activity (i.e., athletic, recreational, or occupational activities requiring physical skills and use strength, power, endurance, speed, flexibility, range of motion, or agility at least 3 days per week) had substantially higher scores on PsyPn than those who did participate in athletic activity. Our results differ from previous research that found athletes respond different to psychosocial health (e.g., disablement, quality of life) constructs (Huffman et al., 2008; McAllister, Motamedi, Hame, Shapiro, & Dorey, 2001); however, they are consistent with previously reported findings, which indicate individuals who are physically active have higher satisfaction with life (Bendíková & Nemček, 2016; Melin, Fugl-Meyer, & Fugl-Meyer, 2003), higher levels of quality of life (Anokye, Trueman, Green, Pavey, & Taylor, 2012), and better psychosocial health outcomes (Dunton, Schneider, & Cooper, 2007; Strine, Chapman, Balluz, Moriarty, & Mokdad, 2008). Therefore, the more active an individual is, the lower the risk for poor psychosocial health outcomes, including PsyPn.

Lastly, differences in variances and means for PsyPn were also found between age groups. In our sample, when comparing total scores for the OMMP-8, the 65+ group had substantially less PsyPn (total score = 13.60) than all other groups (emerging adults = 16.20, early adulthood = 15.99, middle adulthood = 16.57). Our finding is consistent with previous researchers who found that younger individuals exhibit higher levels of PsyPn than older individuals (Orbach et al., 2003; Tossani et al., 2019) and that with age, there is a decrease in psychological distress (Carstensen, Fung, & Charles, 2003). Further, older individuals are more effective and motivated at regulating emotions, particularly disengaging with negative material, which also decreases psychological distress (Rösler et al., 2005; Scheibe & Blanchard-Fields, 2009). Thus, as individuals age, they may report lower scores in PsyPn because there is a decline in frequency and duration of negative emotions and a more positive view on life has developed (Carstensen, Fung, & Charles, 2003; Charles, Mather, & Carstensen, 2003).

#### Latent variable correlational analyses to support construct validity

The OMMP-8 was positively correlated with the PHQ-9 (*r* = .90), DASS-21 (*r* = .86), the subscales of the DASS-21 (*r* = .67 to 84), and negatively correlated with the SCS (*r* = − .85); the findings support the construct validity of the scale (Kline, 2015). The OMMP-8 was also positively correlated with the average NPRS score (*r* = .56). The correlations found in our study align with the multi-factorial definition of PsyPn as measured in the OMMP-8. Additionally, the positive correlations found between the OMMP-8, the DASS-21, and the DASS-21 subscales, are consistent with previous research (Guimarães et al., 2014; Orbach et al., 2003). Although the correlations between the OMMP-8 and the DASS-21 were slightly higher (*r* = .67 to .84) than those previously reported for the OMMP and DASS-21 (Guimarães et al., 2014), our model included three factors, whereas the previous study included five factors of the OMMP. Thus, the reduction in factors and items may have led to the higher correlation value between the scales. More research on the psychometric properties of the OMMP-8, as well as the DASS-21, should be completed to ensure the soundness of the psychometric properties of each scale and to ensure each is measuring a distinguishable experience.

### Clinical implications

Our research identified the OMMP-8 scale (Table 2), which meets strict contemporary measurement criteria, to be recommended for use in research and clinical practice. The OMMP-8 scale met invariance testing recommendations which allows it to be administered in different groups (e.g., males and females, athletes and non-athletes) and allows for group differences to be interpreted as true differences instead of measurement error within the scale (Kline, 2015). Additionally, our findings indicated that respondents with a history of a current or past mental health diagnosis will score higher on the scale. Our results do not support using scores for diagnostic criteria currently; however, they do provide insight into PsyPn and individual well-being, thus positively informing patient care. Lastly, although group comparisons are supported by the invariance testing findings, clinicians and researchers should be cautious using the OMMP-8 to assess change over time until the appropriate analyses (e.g., longitudinal invariance, scale responsiveness) have been completed.

**Table 2:** Orbach and Mikulincer Mental Pain Scale-8

Likert scale: 0: Strongly disagree 1: Disagree 2: Agree to some extent 3: Agree 4: Strongly agree	Strongly disagree	Disagree	Agree to some extent	Agree	Strongly agree
1. The pain will never go away.	O	O	O	O	O
2. I am flooded by many feelings.	O	O	O	O	O
3. I am rejected by everybody.	O	O	O	O	O
4. I will never be able to reduce my pain.	O	O	O	O	O
5. There are strong ups and downs in my feelings.	O	O	O	O	O
6. Nobody is interested in me.	O	O	O	O	O
7. My feelings change all the time.	O	O	O	O	O
8. Others hate me.	O	O	O	O	O

### Limitations and future research

Although our study included a diverse sample, it is not without limitations. The OMMP-8 was assessed using a cross-validation sample with our decision to split the sample; however, the sample used participants who responded to the original 44-item scale. Thus, the responses to the OMMP-8 items could have been influenced by the other 36 items in the scale. Future research should be done on a sample of individuals who only respond to the eight items. Additionally, we found the OMMP-8 was highly correlated with the PHQ-9 and DASS-21. Our findings could indicate refinement of the OMMP led to a more parsimonious scale which had greater overlap with the PHQ-9 and DASS-21. However, conducting similar measurement examination of the DASS-21 and PHQ-9 may also be warranted to ensure those scales meet similar contemporary recommendations and that scale refinement would not alter the resulting correlation values between scales. The psychometric properties of these scales were not assessed in our study and future research should conduct those analyses and re-assess the correlations between scales. Additionally, our findings could have been influenced due to the timing of the scale administration. Data collection occurred at the beginning stages of the COVID-19 pandemic. It is possible that individuals experienced elevated levels of PsyPn, depression, and psychological distress compared to normal, which may have subsequently impacted participant responses and the correlation values found between scales.

Although the OMMP-8 is a more parsimonious scale to assess PsyPn, more work should be done to validate the scale structure in new samples. More research should be performed with adolescents, as the rates in suicide have increased in this demographic dramatically (World Health Organization, 2019). Additionally, because it may be important for clinicians and researchers to assess change over time, reliability, responsiveness, minimal clinically important differences, and longitudinal invariance analyses should be conducted to ensure that the measurement properties of the scale are invariant over time (Kline, 2015). Lastly, we must consider the purpose and utilization of this scale. The OMMP was designed as a comprehensive instrument to assess the unique constructs of PsyPn. While participant scores on the OMMP-8 were highly correlated (*r* = .925) with the original OMMP, the elimination of so many items and factors should be reviewed to ensure the refined tool captures the desired multi-factorial nature of PsyPn. Researchers may want to consider conducting further analyses that correlate OMMP-8 responses (sub-dimensions and higher order latent variables) with other scales designed to measure relevant factors of PsyPn. Researchers may also want to consider adding novel items to tap into sub-constructs of PsyPn that are not captured in the OMMP-8. In particular, rewriting items to capture the respondent experience of “Emptiness” and “Loss of Control” should be examined because researchers have found individuals who attempt suicide score significantly higher in these dimensions (Levi-Belz et al., 2017).

## Conclusions

The original scale structure of the OMMP was not supported in our study. We subsequently identified a refined 3-factor, 8-item OMMP (i.e., OMMP-8) that met contemporary recommendations for model fit and multi-group invariance testing. Our findings support the OMMP-8 as a more viable option to assess PsyPn in research and clinical practice, but caution is warranted until more research is completed to further assess the measurement properties of the refined scale.

## Supplementary Information

**Additional file 1: Supplemental Table 1** Exploratory Factor Analysis Solutions Validating the OMMP. **Supplemental Table 2** Cronbach’s Alpha Across Samples. **Supplemental Table 3** Correlations Between First-Order Latent Variables OMMP. **Supplemental Table 4** Initial Exploratory Factor Analysis OMMP. **Supplemental Table 5** Parallel Analysis Raw Data Eigenvalues, Means and Percentile Random Data Eigenvalues. **Supplemental Table 6** Refined OMMP-9 Exploratory Factor Analysis. **Supplemental Table 7** Goodness-of-fit Indices for Measurement Invariance Analyses Across Mental Health Diagnoses OMMP-8. **Supplemental Table 8** Goodness-of-fit Indices for Measurement Invariance Analyses Across Sex OMMP-8. **Supplemental Table 9** Goodness-of-fit Indices for Measurement Invariance Analyses Across Injury Status. **Supplemental Table 10** Goodness-of-fit Indices for Measurement Invariance Analyses Across Age Groups. **Supplemental Table 11** Goodness-of-fit Indices for Measurement Invariance Analyses Across Activity Level. **Supplemental Table 12** Goodness-of-fit Indices for Measurement Invariance Analyses Across Athletic Classification. **Supplemental Figure 1** Covariance Model OMMP-9.

## Data Availability

Datasets used and analyzed are available from the corresponding author upon reasonable request.
