# Supplementary material for: Measuring psychological pain: psychometric analysis of the Orbach and Mikulincer Mental Pain Scale
Source: Meas Instrum Soc Sci. 2021 May 17;3(1):7. doi: 10.1186/s42409-021-00025-8 (PMC8127506; doi:10.1186/s42409-021-00025-8)
Supplement: Supplementary file 1 — Additional file 1: Supplemental Table 1 Exploratory Factor Analysis Solutions Validating the OMMP. Supplemental Table 2 Cronbach’s Alpha Across Samples. Supplemental Table 3 Correlations Between First-Order Latent Variables OMMP. Supplemental Table 4 Initial Exploratory Factor Analysis OMMP. Supplemental Table 5 Parallel Analysis Raw Data Eigenvalues, Means and Percentile Random Data Eigenvalues. Supplemental Table 6 Refined OMMP-9 Exploratory Factor Analysis. Supplemental Table 7 Goodness-of-fit Indices for Measurement Invariance Analyses Across Mental Health Diagnoses OMMP-8. Supplemental Table 8 Goodness-of-fit Indices for Measurement Invariance Analyses Across Sex OMMP-8. Supplemental Table 9 Goodness-of-fit Indices for Measurement Invariance Analyses Across Injury Status. Supplemental Table 10 Goodness-of-fit Indices for Measurement Invariance Analyses Across Age Groups. Supplemental Table 11 Goodness-of-fit Indices for Measurement Invariance Analyses Across Activity Level. Supplemental Table 12 Goodness-of-fit Indices for Measurement Invariance Analyses Across Athletic Classification. Supplemental Figure 1 Covariance Model OMMP-9. [file 42409_2021_25_MOESM1_ESM.docx]

**Supplemental Tables and Figures**

**Supplemental Table 1**

*Exploratory Factor Analysis Solutions Validating the OMMP*

| **Subjects** | **Sample 1** | **Sample 2** | **Sample 3** | **Sample 4** | **Sample 5** |
| --- | --- | --- | --- | --- | --- |
| *Item* | *Factor* | *Factor* | *Factor* | *Factor* | *Factor* |
| 10. I have lost something that I will never find again. | IR | IR | IR | IR | IR |
| 44. The pain will never go away. | IR | IR | IR | IR | *DNF* |
| 22. The difficult situation will never change. | IR | IR | *DNF* | *DNF* | *DNF* |
| 26. The world has changed forever. | IR | IR | IR | *DNF* | IR |
| 30. My life has stopped. | IR | IR | *DNF* | *DNF* | *DNF* |
| 32. Something in my life was damaged forever. | IR | IR | IR | IR | IR |
| 34. I will never be the same person. | IR | IR | IR | *DNF* | IR |
| 43. I can’t change what is happening to me. | IR | IR | EMP | *DNF* | *DNF* |
| 29. I will never be able to reduce my pain. | IR | IR | EMP | IR | *DNF* |
| 6. I am afraid of the future. | LC | LC/FRZ | *DNF* | *DNF* | *DNF* |
| 36. I have no control over the situation. | LC | *DNF* | *DNF* | *DNF* | IR |
| 33. There is uncertainty about my life and myself. | LC | LC/FRZ | IR | *DNF* | IR |
| 31. I have no idea what to expect of the future. | LC | LC/FRZ | *DNF* | *DNF* | *DNF* |
| 13. I have no control over my life. | LC | *DNF* | *DNF* | *DNF* | *DNF* |
| 2. I am completely helpless. | LC | *DNF* | HP | LC | HP |
| 28. I have no control over what is happening inside me. | LC | *DNF* | EF | *DNF* | POW |
| 9. I am completely defeated. | LC | *DNF* | *DNF* | *DNF* | *DNF* |
| 5. I will fall apart. | LC | LC/FRZ | HP | LC | HP |
| 21. I cannot trust myself. | LC | LC/FRZ | *DNF* | LC | POW |
| 7. I am rejected by everybody. | NW | NW | *DNF* | NW | HP |
| 12. I feel abandoned and lonely. | NW | NW | HP | *DNF* | *DNF* |
| 1. Nobody is interested in me. | NW | NW | HP | NW | *DNF* |
| 16. Others hate me. | NW | NW | *DNF* | NW | *DNF* |
| 18. I am worthless. | NW | NW | *DNF* | *DNF* | *DNF* |
| 14. My feelings change all the time. | EF | EF | EF | EF | EF |
| 35. There are strong ups and downs in my feelings. | EF | EF | EF | EF | EF |
| 3. I feel an emotional turmoil inside me. | EF | LC/FRZ | EF | EF | *DNF* |
| 8. I am flooded by many feelings. | FRZ | EF | EF | EF | EF |
| 11. I feel numb and not alive. | FRZ | LC/FRZ | *DNF* | *DNF* | *DNF* |
| 19. I feel paralyzed | FRZ | LC/FRZ | *DNF* | LC | *DNF* |
| 4. I cannot do anything at all | EST | LC/FRZ | HP | LC | HP |
| 17. I feel that I am not my old self anymore. | EST | *DNF* | *DNF* | *DNF* | *DNF* |
| 23. I feel as if I am not real. | EST | *DNF* | CON | *DNF* | *DNF* |
| 15. I am a stranger to myself. | EST | NW | *DNF* | *DNF* | POW |
| 20. I cannot concentrate. | CON | LC/FRZ | CON | LC | *DNF* |
| 24. I have difficulties in thinking. | CON | *DNF* | CON | LC | POW |
| 27. I feel confused. | CON | *DNF* | *DNF* | *DNF* | *DNF* |
| 37. I want to be left alone. | SD | *NI* | *NI* | *NI* | *NI* |
| 25. I need the support of other people. ® | SD | *NI* | *NI* | *NI* | *NI* |
| 40. I don’t feel like talking to other people. | SD | *NI* | *NI* | *NI* | *NI* |
| 42. I can’t stay alone. ® | SD | *NI* | *NI* | *NI* | *NI* |
| 41. I can’t find meaning in my life. | EMP | EMP | EMP | EMP | EMP |
| 39. I have no desires | EMP | EMP | EMP | EMP | EMP |
| 38. I have no future goals. | EMP | EMP | EMP | EMP | EMP |
| Factor Names: IR = Experience of irreversibility; LC = Loss of control; NW = Narcissistic wounds; EF = Emotional flooding; FRZ = Freezing; EST = Estrangement; CON = Confusion; SD = Social distancing; EMP = Emptiness; LC/FRZ = Lack of Control and Freezing; HP = Helplessness; POW = Powerlessness. Other Abbreviations and Symbols: NI = Item Not Included in Analysis; DNF = Item Did Not Factor. Sample 1 = 513 Israeli Jewish adults (Orbach et al., 2003); Sample 2 = 544 Italian adults (Tossani et al., 2019); Sample 3 = 403 drug addicted adults (Guimarães et al., 2014); Sample 4 = 427 Korean students (Heo, 2008); Sample 5 = 229 US students (Heo, 2008). | | | | | |

**Supplemental Table 2**

*Cronbach’s Alpha Across Samples*

| **Factors** | **Sample 1** | **Sample 2** | **Sample 3** | **Sample 4** | **Sample 5** |
| --- | --- | --- | --- | --- | --- |
| Experience of irreversibility* | .95 | .90 | .82 | .81 | .89 |
| Loss of control* | .95 | - | - | .90 | - |
| Narcissistic wounds* | .93 | .86 | - | .81 | - |
| Emotional flooding* | .93 | .85 | .80 | .88 | .80 |
| Freezing* | .85 | - | - | - | - |
| Estrangement* | .79 | *-* | - | - | - |
| Confusion* | .80 | *-* | .75 | - | - |
| Social distancing* | .80 | *-* | - | - | - |
| Emptiness* | .75 | .81 | .83 | .82 | .87 |
| Lack of Control and Freezing |  | .92 |  |  |  |
| Helplessness |  |  | .78 |  | .91 |
| Powerlessness |  |  |  |  | .88 |
| * indicates one of the original nine factors; Sample 1 = 513 Israeli Jewish adults (Orbach et al., 2003); Sample 2 = 544 Italian adults (Tossani et al., 2019); Sample 3 = 403 drug addicted adults (Guimarães et al., 2014); Sample 4 = 427 Korean students (Heo, 2008); Sample 5 = 229 US students (Heo, 2008). | | | | | |

**Supplemental Table 3**

*Correlations Between First-Order Latent Variables OMMP*

| **Factors** | **IRR** | **LOSS** | **NW** | **EF** | **FRZ** | **EST** | **CONF** | **SD** | **EMP** |  |
| --- | --- | --- | --- | --- | --- | --- | --- | --- | --- | --- |
| IRR | 1.0 |  |  |  |  |  |  |  |  |  |
| LOSS | .89 | 1.0 |  |  |  |  |  |  |  |  |
| NW | .80 | .89 | 1.0 |  |  |  |  |  |  |  |
| EF | .71 | .82 | .66 | 1.0 |  |  |  |  |  |  |
| FRZ | .83 | .94 | .92 | .67 | 1.0 |  |  |  |  |  |
| EST | .84 | .91 | .89 | .77 | .90 | 1.0 |  |  |  |  |
| CONF | .76 | .88 | .69 | .84 | .78 | .83 | 1.0 |  |  |  |
| SD | .67 | .71 | .72 | .66 | .69 | .76 | .71 | 1.0 |  |  |
| EMP | .77 | .75 | .76 | .52 | .79 | .79 | .61 | .71 | 1.0 |  |
| IRR = Experience of irreversibility; LOSS = Loss of control; NW = Narcissistic wounds; EF = Emotional flooding; FRZ = Freezing; EST = Estrangement; CONF = Confusion; SD = Social distancing; EMP = Emptiness | | | | | | | | | | |

**Supplemental Table 4**

*Initial Exploratory Factor Analysis OMMP*

| **Item** | **Factor 1** | **Factor 2** | **Factor 3** | **Factor 4** |
| --- | --- | --- | --- | --- |
| 4. I cannot do anything at all. | **.812** | .050 | .068 | -.075 |
| 2. I am completely helpless. | **.773** | -.058 | -.099 | -.033 |
| 9. I am completely defeated. | **.768** | .044 | -.080 | -.037 |
| 18. I am worthless. | **.756** | -.095 | -.166 | .111 |
| 19. I feel paralyzed. | **.651** | .132 | -.036 | .072 |
| 21. I cannot trust myself. | **.616** | .233 | .061 | .083 |
| 11. I feel numb and not alive. | **.599** | .052 | -.122 | .197 |
| 7. I am rejected by everybody. | **.561** | .040 | .000 | .250 |
| 5. I will fall apart. | **.559** | .314 | -.012 | -.005 |
| 30. My life has stopped. | **.554** | .071 | -.273 | -.072 |
| 13. I have no control over my life. | **.536** | .240 | -.093 | -.019 |
| 22. The difficult situation will never change. | **.508** | .124 | -.317 | -.048 |
| 41. I can’t find meaning in my life. | **.481** | .049 | -.178 | .314 |
| 23. I feel as if I am not real. | **.474** | .077 | -.107 | .175 |
| 15. I am a stranger to myself. | **.468** | .119 | -.056 | .297 |
| 39. I have no desires. | **.464** | -.152 | -.239 | .326 |
| 12. I feel abandoned and lonely. | **.460** | .107 | -.173 | .222 |
| 38. I have no future goals. | **.420** | -.177 | -.321 | .295 |
| 1. Nobody is interested in me. | **.419** | .007 | .006 | .365 |
| 16. Others hate me. | **.398** | .097 | -.077 | .229 |
| 28. I have no control over what is happening inside me. | **.392** | .313 | -.227 | .023 |
| 8. I am flooded by many feelings. | .032 | **.747** | .044 | .097 |
| 35. There are strong ups and downs in my feelings. | -.079 | **.708** | -.156 | .159 |
| 27. I feel confused. | .280 | **.637** | .069 | .011 |
| 3. I feel an emotional turmoil inside me. | .138 | **.636** | -.018 | .135 |
| 14. My feelings change all the time. | .075 | **.621** | .031 | .123 |
| 25. I need the support of other people. ® | .072 | **-.592** | .054 | .192 |
| 33. There is uncertainty about my life and myself. | .084 | **.568** | -.161 | .128 |
| 20. I cannot concentrate. | .244 | **.559** | .099 | .147 |
| 26. The world has changed forever. | -.056 | **.548** | -.086 | .026 |
| 31. I have no idea what to expect of the future. | .192 | **.528** | -.053 | .080 |
| 34. I will never be the same person. | -.212 | **.502** | -.376 | .239 |
| 6. I am afraid of the future. | .358 | **.490** | .133 | .064 |
| 24. I have difficulties in thinking. | .247 | **.477** | -.021 | .151 |
| 42. I can’t stay alone. ® | -.234 | **-.430** | .040 | .104 |
| 17. I feel that I am not my old self anymore. | .012 | **.375** | -.182 | .295 |
| 36. I have no control over the situation. | .314 | **.329** | -.269 | -.139 |
| 44. The pain will never go away. | .089 | -.023 | **-.847** | .001 |
| 29. I will never be able to reduce my pain. | .227 | -.018 | **-.771** | -.056 |
| 43. I can’t change what is happening to me. | .291 | .240 | **-.464** | -.104 |
| 32. Something in my life was damaged forever. | .004 | .312 | **-.453** | .200 |
| 10. I have lost something that I will never find again. | .005 | .243 | **-.375** | .222 |
| 37. I want to be left alone. | .061 | .155 | -.017 | **.631** |
| 40. I don’t feel like talking to other people. | .153 | .126 | -.055 | **.598** |

**Supplemental Table 5**

*Parallel Analysis Raw Data Eigenvalues, Means and Percentile Random Data Eigenvalues*

| **Number of items** | **Raw Data** | **Means** | **Random Data** |
| --- | --- | --- | --- |
| 1 | *20.71** | *1.57* | *1.63** |
| 2 | *2.73** | *1.51* | *1.55** |
| 3 | *1.64** | *1.46* | *1.50** |
| 4 | *1.48** | *1.43* | *1.46** |
| 5 | .974 | 1.39 | 1.42 |
| 6 | .923 | 1.36 | 1.39 |
| 7 | .854 | 1.33 | 1.36 |
| 8 | .833 | 1.30 | 1.33 |
| 9 | .783 | 1.27 | 1.30 |

* p < .05

*Note: Table only presents data for the first 9 of 44 items.*

**Supplemental Table 6**

*Refined OMMP-9 Exploratory Factor Analysis*

| **Item** | **1** | **2** | **3** |
| --- | --- | --- | --- |
| 44. The pain will never go away. | .957 |  |  |
| 29. I will never be able to reduce my pain. | .855 |  |  |
| 32. Something in my life was damaged forever. | .425 |  |  |
| 8. I am flooded by many feelings. |  | .847 |  |
| 35. There are strong ups and downs in my feelings. |  | .826 |  |
| 14. My feelings change all the time. |  | .763 |  |
| 7. I am rejected by everybody. |  |  | .857 |
| 1. Nobody is interested in me. |  |  | .715 |
| 16. Others hate me. |  |  | .600 |
| **Eigenvalues** | **4.54** | **1.22** | **1.02** |
| **% of variance** | **50.48** | **13.56** | **11.34** |
| **Cronbach’s alpha** | **.835** | **.856** | **.767** |

**Supplemental Table 7**

*Goodness-of-fit Indices for Measurement Invariance Analyses Across Mental Health Diagnoses OMMP-8*

|  | *χ^2^* | df | *χ^2^*_diff_ (df_diff_) | CFI | CFI_diff_ | TLI | RMSEA |
| --- | --- | --- | --- | --- | --- | --- | --- |
| Mental health diagnosis  (n = 396) | 12.69 | 17 | ---- | 1.00 | ---- | 1.00 | .000 |
| No mental health diagnosis  (n = 633) | 65.89 | 17 | ---- | .979 | ---- | .965 | .067 |
| Configural (equal form) | 78.56 | 34 | ---- | .988 | ---- | .980 | .036 |
| Metric (equal loadings) | 83.30 | 39 | 4.74(5) | .988 | <.001 | .983 | .033 |
| Equal factor variances | 122.23 | 42 | **43.67(8)** | .978 | .01 | .978 | .043 |
| Scalar  (equal indicator intercepts) | 100.20 | 44 | 21.64(10) | .985 | .003 | .981 | .035 |
| Equal latent means | 208.16 | 47 | **129.60(13)** | .956 | **.032** | .948 | .058 |

**Supplemental Table 8**

*Goodness-of-fit Indices for Measurement Invariance Analyses Across Sex OMMP-8*

|  | *χ^2^* | df | *χ^2^*_diff_ (df_diff_) | CFI | CFI_diff_ | TLI | RMSEA |
| --- | --- | --- | --- | --- | --- | --- | --- |
| Males (n = 206) | 40.54 | 17 | ---- | .970 | ---- | .951 | .082 |
| Females (n = 833) | 43.52 | 17 | ---- | .992 | ---- | .986 | .043 |
| Configural (equal form) | 84.15 | 34 | ---- | .987 | ---- | .979 | .038 |
| Metric (equal loadings) | 86.61 | 39 | 2.46(5) | .988 | +.001 | .983 | .034 |
| Equal factor Variances | 89.75 | 42 | 5.60(8) | .988 | +.001 | .984 | .033 |
| Scalar  (equal indicator intercepts) | 101.13 | 44 | 16.99(10) | .985 | .002 | .981 | .035 |
| Equal latent means | 132.68 | 47 | **48.53(13)** | .978 | .009 | .974 | .042 |

**Supplemental Table 9**

*Goodness-of-fit Indices for Measurement Invariance Analyses Across Injury Status*

|  | *χ^2^* | df | *χ^2^*_diff_ (df_diff_) | CFI | CFI_diff_ | TLI | RMSEA |
| --- | --- | --- | --- | --- | --- | --- | --- |
| Healthy (n = 662) | 36.94 | 17 | ---- | .992 | ---- | .986 | .042 |
| Injured (n = 388) | 22.55 | 17 | ---- | .996 | ---- | .994 | .029 |
| Configural (equal form) | 59.49 | 34 | ---- | .993 | ---- | .989 | .027 |
| Metric (equal loadings) | 63.28 | 39 | 3.79(5) | .994 | +.001 | .991 | .024 |
| Equal factor variances | 190.45 | 42 | **130.96(8)** | .961 | **.021** | .948 | .058 |
| Scalar  (equal indicator intercepts) | 72.40 | 44 | 12.91(10) | .993 | NC | .991 | .025 |
| Equal latent means | 222.23 | 47 | **162.74(13)** | .954 | **.032** | .945 | .060 |

**Supplemental Table 10**

*Goodness-of-fit Indices for Measurement Invariance Analyses Across Age Groups*

|  | *χ^2^* | df | *χ^2^*_diff_ (df_diff_) | CFI | CFI_diff_ | TLI | RMSEA |
| --- | --- | --- | --- | --- | --- | --- | --- |
| Emerging Adulthood (n=211) | 33.61 | 17 | ---- | .980 | ---- | .967 | .068 |
| Early Adulthood (n=388) | 7.54 | 17 | ---- | 1.00 | ---- | 1.00 | .000 |
| Middle Adulthood (n=334) | 32.87 | 17 | ---- | .988 | ---- | .980 | .053 |
| Late Adulthood (n = 114) | 22.04 | 17 | ---- | .983 | ---- | .973 | .051 |
| Configural (equal form) | 96.16 | 68 | ---- | .993 | ---- | .988 | .020 |
| Metric (equal loadings) | 123.78 | 83 | 27.62(15) | .993 | .003 | .986 | .022 |
| Equal factor variances) | 230.63 | 92 | **134.47(24)** | .964 | **.029** | .957 | .038 |
| Scalar  (equal indicator intercepts) | 168.23 | 98 | 72.07(30) | .982 | .011 | .979 | .026 |
| Equal latent means | 341.65 | 107 | **245.49(39)** | .940 | **.053** | .937 | .046 |

**Supplemental Table 11**

*Goodness-of-fit Indices for Measurement Invariance Analyses Across Activity Level*

|  | *χ^2^* | df | *χ^2^*_diff_ (df_diff_) | CFI | CFI_diff_ | TLI | RMSEA |
| --- | --- | --- | --- | --- | --- | --- | --- |
| Inactive/Low (n = 589) | 33.43 | 17 | ---- | .992 | ---- | .987 | .041 |
| Moderate/High (n = 461) | 17.52 | 17 | ---- | 1.00 | ---- | .999 | .008 |
| Configural (equal form) | 50.94 | 34 | ---- | .995 | ---- | .993 | .022 |
| Metric (equal loadings) | 55.33 | 39 | 4.39(5) | .996 | +.001 | .994 | .020 |
| Equal factor variances | 117.11 | 42 | 66.17(8) | .980 | **.015** | .973 | .041 |
| Scalar  (equal indicator intercepts) | 62.75 | 44 | 11.81(10) | .995 | NC | .994 | .020 |
| Equal latent means | 145.27 | 47 | 94.33(13) | .974 | **.021** | .969 | .045 |

**Supplemental Table 12**

*Goodness-of-fit Indices for Measurement Invariance Analyses Across Athletic Classification*

|  | *χ^2^* | df | *χ^2^*_diff_ (df_diff_) | CFI | CFI_diff_ | TLI | RMSEA |
| --- | --- | --- | --- | --- | --- | --- | --- |
| Athletic activity (n = 455) | 27.96 | 17 | ---- | .993 | ---- | .989 | .038 |
| No athletic activity (n = 595) | 40.14 | 17 | ---- | .989 | ---- | .982 | .048 |
| Configural (equal form) | 68.13 | 34 | ---- | .991 | ---- | .985 | .031 |
| Metric (equal loadings) | 72.16 | 39 | 4.03(5) | .991 | NC | .987 | .028 |
| Equal factor variances | 116.38 | 42 | **48.25(8)** | .980 | **.011** | .974 | .041 |
| Scalar  (equal indicator intercepts) | 82.58 | 44 | 14.45(10) | .990 | .001 | .987 | .029 |
| Equal latent means | 154.13 | 47 | **86.0(13)** | .972 | **.019** | .966 | .047 |

**Supplemental Figure 1**

*Covariance Model OMMP-9*

**
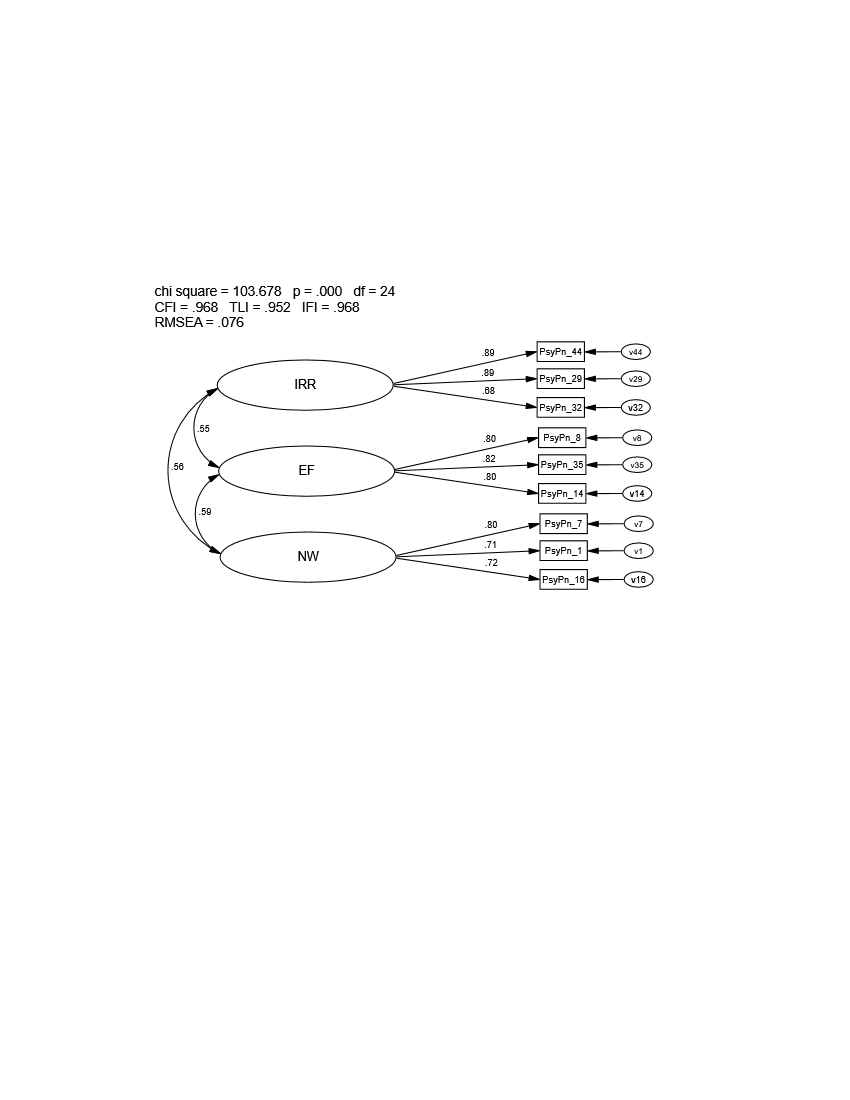
**
